# Mediastinal emphysema after long‐distance flight with ketoacidosis and underlying diabetes mellitus type 1

**DOI:** 10.1002/rcr2.423

**Published:** 2019-04-05

**Authors:** Gracia Lana Ardila Pardo, Wolfgang Michael Kübler, Martin Witzenrath, Jörg‐Wilhelm Oestmann

**Affiliations:** ^1^ Department of Radiology Charité Universitätsmedizin Berlin Berlin Germany

**Keywords:** Diabetes mellitus type 1, ketoacidosis, long‐distance flight, mediastinal emphysema, pneumomediastinum

## Abstract

A 21‐year old female with diabetes mellitus type 1 presented to our hospital's emergency department having suffered from shortness of breath, mild chest pain, and vomiting following her arrival after a long‐distance flight two days earlier. Symptoms had since subsided and physical examination was normal. Blood analysis revealed increased D‐dimers and diabetic ketoacidosis. Computed tomography (CT) examination excluded pulmonary embolism but demonstrated significant mediastinal emphysema. After conservative treatment including nasal oxygen and adjustment of insulin therapy, follow‐up low‐dose CT after four days confirmed full regression of the emphysema. The patient was discharged feeling well, with a recommendation for improved diabetes treatment. Spontaneous pneumomediastinum is a rare condition occurring in younger patients without trauma or pulmonary disease. Over‐inflation and/or pulmonary vasoconstriction have been proposed as major physiological contributors and were likely evoked in the present case by increased respiratory drive due to ketoacidosis and hypoxic vasoconstriction during long distance flight.

## Introduction

Spontaneous pneumomediastinum (SPM) is a rare differential diagnosis of acute chest pain and dyspnoea. Clinical symptoms may be subtle. A number of risk factors increase the chance of SPM. SPM normally regresses spontaneously within a few days.

## Case Report

A 21‐year‐old female Caucasian presented two days after a 24 h long‐distance flight. On the morning after the flight, shortness of breath, thoracic tenderness, and overall physical discomfort developed. Tenderness increased the next day. Clinical examinations revealed good general condition/nutritional status (body mass index of 18.7), with no signs of dyspnoea or fever. Blood tests revealed acidosis (pH: 7.15; reference value: 7.26–7.46), and hyperglycaemia (glucose: 418 mg/dL; pre‐prandial reference value: 90–120 mg/dL) congruent with diabetic ketoacidosis. Electrocardiogram was unremarkable.

**Figure 1:**
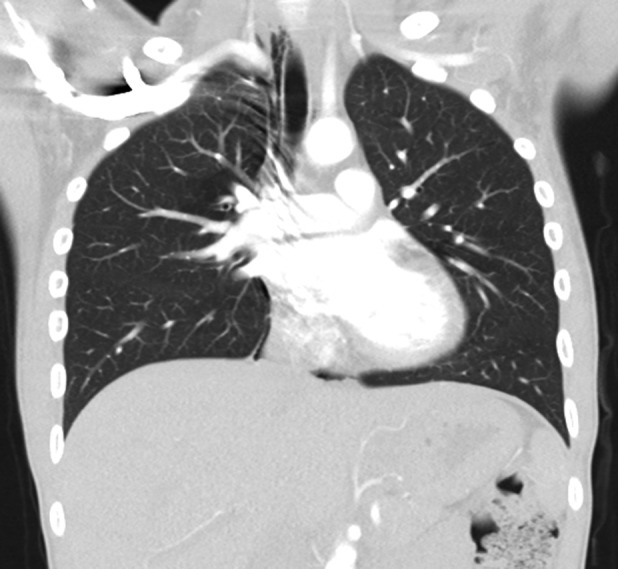
Coronal computed tomography scan (Toshiba Aquilion 64, acquisition parameters: 120 kV, 20 mA) shows a significant mediastinal emphysema more pronounced on the right side.

Computed tomography (CT) excluded pulmonary embolism but demonstrated mediastinal emphysema (Fig. 1). Therapy started with nasal oxygen and corrective insulin dosage. Subsequently, pH in venous blood increased to 7.294 at a standard base excess of −11.8 mmoL/L (−2 to +3 mmoL/L), standard bicarbonate of 15.5 mmoL/L (21–26 mmoL/L), partial pressure of carbon dioxide (pCO_2_) of 28.5 mmHg (41–51 mmHg), partial pressure of oxygen (pO_2_) of 52.9 mmHg (20–49 mmHg), and saturated oxygen (sO_2_) of 87.5% (70–75%). Glucose decreased to 354 mg/dL. Haemoglobin A1c at 13.2% (<5.7%; therapeutic target value in adults with diabetes mellitus type 1 (DMT1) < 7.5%) indicated chronic diabetic derailment. D‐dimers were increased at 0.83 μg/mL (<0.5 μg/mL).

**Figure 2:**
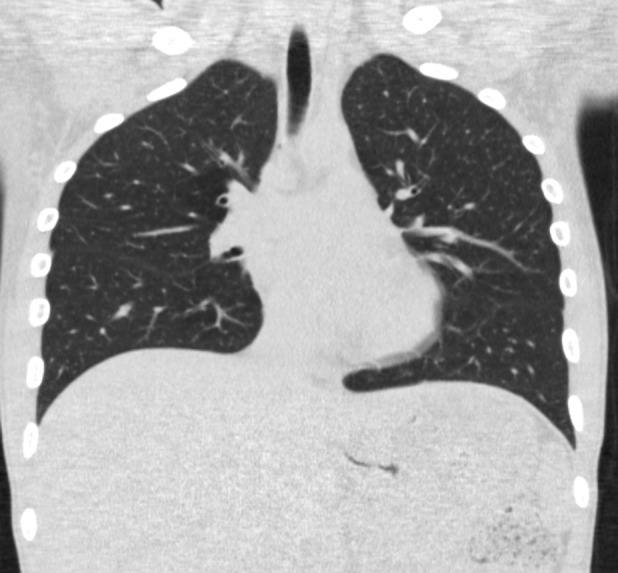
Coronal computed tomography scan, low dose (Toshiba Aquilion 64, acquisition parameters: 120 kV, 20 mA) shows full regression of the mediastinal emphysema.

CT four days later showed full resolution of mediastinal emphysema (Fig. 2).

Further inquiry revealed no specific events during or before the flights (two stopovers, no elevator rides). No nausea, vomiting, coughing, or ear pain was noted during the flight or immediately thereafter. Valsalva manoeuvres were not performed.

DMT1 had been diagnosed eight years earlier and treated thereafter with insulin glargine and lispro. Blood glucose profiles had been sub‐optimal, but no symptoms arose. Other/previous illnesses and smoking were denied.

The patient was discharged on the fifth day. Diabetological supervision was recommended.

## Discussion

In pneumomediastinum air leaks into the mediastinum either directly or via the pulmonary interstitium 1. As opposed to non‐spontaneous pneumomediastinum (NSPM) SPM lacks an obvious cause 2. In most cases SPM is self‐limiting without serious consequences 2, 3.

Patients typically are young men presenting with sharp thoracic and retrosternal pain, dyspnoea, neck and back pain, subcutaneous emphysema, coughing, or dysphagia 2, 4, 5, 6. “Hamman's sign,” a rasping systolic sound, can sometimes be auscultated precordially 7.

Acute coronary syndrome, pericarditis, and Boerhaave's syndrome should be excluded on clinical grounds 3, and NSPM, pneumothorax, pulmonary embolism, and pericardiac effusion by CT scan 2, 3. A severe form of SPM with shock, dyspnoea, and cyanosis may simulate heart disease 8.

In uncomplicated cases, pain therapy suffices 2. If significant symptoms arise, monitoring and oxygen application is necessary.

**Figure 3:**
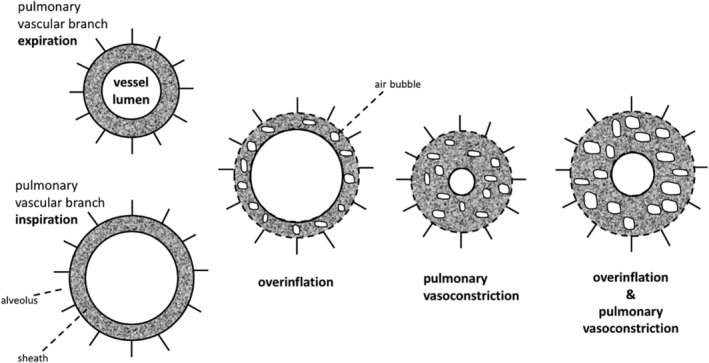
The model by Macklin and Macklin. The authors coined alveolar over‐expansion as Factor A, while vasoconstriction was termed Factor B. As both factors apply, the vascular sheath widens and in the process facilitates and readily accepts air leakage into the interstitium.

The pathogenesis of SPM was outlined in detail by Macklin and Macklin as a state of increased mediastinal pressure 8. According to this concept, the following two factors may cause rupture of the alveolar membrane and subsequent air leak (Fig. 3).

Alveolar over‐inflation with subsequent alveolar over‐inflation and rupture with consecutive transfer of air along the bronchi into the mediastinum has been identified as primary mechanism 5. As such, increased intra‐alveolar pressure (relative to the interstitium surrounding peripheral alveoli), during coughing, vomiting, and cocaine sniffing, and barotrauma during diving or gas inhalation present characteristic triggers of SPM 9, 10, 11, 12, 13, 14. Similarly, activities associated with alveolar over‐inflation such as pronounced physical activity have been associated with SPM 15. It is thus little surprising that multiple reports have found a co‐existence of SPM and ketoacidosis 11, 16, 17, 18, 19, which has been attributed to diabetic hyperventilation (Kussmaul's respiration) triggered by increased respiratory drive at low arterial pH 11, 16, 17, 18, 19.

Vasoconstriction, either acting alone or in combination with alveolar over‐inflation, may increase strain on adjacent marginal alveoli, thereby causing over‐distension and air leak into the perivascular sheath and, ultimately, the mediastinum 8. In the present case, it is tempting to speculate that hypoxic pulmonary vasoconstriction during the long‐distance flight, where cabin pressure typically reaches 550 mmHg (equivalent to an altitude of 2500 m) equivalent to an inspired oxygen fraction (FiO_2_) at sea level of 15%, contributed to SPM.

When first described radiologically in 2007, SPM during flight was attributed to increased relative intra‐alveolar pressure due to transient/chronic air trapping at high altitude 20. In the present case, however, no signs of air trapping were detected.

In line with the concept by Macklin and Macklin, SPM in the present case was thus likely brought about by a synergistic, over‐additive combination of alveolar over‐inflation and pulmonary vasoconstriction. To the best of our knowledge this is the first published case presenting a combination of both mechanisms as a likely cause of SPM.

### Disclosure Statement

Appropriate written informed consent was obtained for publication of this case report and accompanying images.
