# Corrigendum

**DOI:** 10.1002/rcr2.449

**Published:** 2019-06-12

The authors would like to draw the reader's attention to two errors in the following article:

Ardila Pardo, GL, Kübler, WM, Witzenrath, M, Oestmann, J. (2019) Mediastinal emphysema after long‐distance flight with ketoacidosis and underlying diabetes mellitus type 1. Respirology Case Reports, 7(5),; e00423. https://doi.org/10.1002/rcr2.423

1. Prof Wolfgang Michael Kübler's last name was misspelled.

The correct spelling is: Wolfgang Michael Kuebler

2. In Figure 1 the acquisition parameters were: 100 kV, 147 mA.

The authors apologize for these errors and any confusion it may have caused.

